# Association of Major Histocompatibility Complex Class I Related Chain A/B Positive Microparticles with Acute Myocardial Infarction and Disease Severity

**DOI:** 10.3390/diagnostics10100766

**Published:** 2020-09-29

**Authors:** Songpol Haohan, Burabha Pussadhamma, Amonrat Jumnainsong, Wit Leuangwatthananon, Pattarapong Makarawate, Chanvit Leelayuwat, Nantarat Komanasin

**Affiliations:** 1 Cardiovascular Research Group, Khon Kaen University, Khon Kaen 40002, Thailand; songpol_ams@hotmail.com (S.H.); pussadhamma@gmail.com (B.P.); pong_pattarapong@hotmail.com (P.M.); 2 Biomedical Sciences Program, Graduate School, Khon Kaen University, Khon Kaen 40002, Thailand; 3 Division of Cardiology, Department of Medicine, Faculty of Medicine, Khon Kaen University, Khon Kaen 40002, Thailand; 4 School of Medical Technology, Faculty of Associated Medical Sciences, Khon Kaen University, Khon Kaen 40002, Thailand; amonrat@kku.ac.th (A.J.); chanvit@kku.ac.th (C.L.); 5 The Centre for Research and Development of Medical Diagnostic Laboratories (CMDL), Faculty of Associated Medical Sciences, Khon Kaen University, Khon Kaen 40002, Thailand; 6 Queen Sirikit Heart Center of the Northeast Hospital, Khon Kaen University, Khon Kaen 40002, Thailand; witz_yellow@hotmail.com

**Keywords:** MICA/B^+^ MPs, microparticles, acute myocardial infarction, NKG2D, atherosclerosis, inflammation

## Abstract

Background: Various cell types undergo activation and stress during atherosclerosis resulting in the development of acute myocardial infarction (AMI) in coronary artery disease (CAD). Major histocompatibility complex class I related chain A and B (MICA/B) can be expressed on the surface of activated and stressed cells and released into blood circulation in several forms including microparticles (MICA/B^+^ MPs) from various cell types. We aimed to investigate the association of these MICA/B^+^ MPs with the presence of AMI. Fifty-one AMI and 46 age-matched control subjects were recruited. Methods: Levels of MICA/B^+^ MPs derived from various parent cells including endothelial cells, platelets, monocytes, neutrophils, and T lymphocytes were determined by flow cytometry. Results: The levels and proportion of MICA/B^+^ MPs from all types of cell origin were significantly increased in AMI patients compared to those of the controls. A multivariate regression model showed an independent association between MICA/B^+^ MPs and AMI (OR = 11.6; 95% CI = 2.8, 47.3). Interestingly, based on the disease severity, we found that the levels of MICA/B^+^ MPs were significantly elevated in the ST-segment elevation myocardial infarction (STEMI) compared to the non-STEMI (NSTEMI) patients. Moreover, an independent association of MICA/B^+^ MPs with the occurrence of STEMI was also demonstrated (OR = 4.1; 95% CI = 1.5, 16.7). Conclusions: These results suggest that MICA/B^+^ MPs are associated with AMI and disease severity. They may act as mediators contributing to the pathological process of AMI. Alternatively, they are the results of various cell activations contributing to AMI.

## 1. Introduction

Major histocompatibility complex class I related chain A and B (MICA/B) are major ligands of the natural killer group 2, member D (NKG2D) activating receptor, which is normally expressed on many immune cells including natural killer (NK) cells, natural killer T (NKT) cells, CD8 T cells, and γδ T cells [1]. The interaction between NKG2D and its ligands is reported to promote inflammation by releasing many cytokines. It also enhances the cytotoxic function of immune cells. MICA/B are stress-induced proteins and are associated with various inflammatory and autoimmune diseases such as rheumatoid arthritis, colitis, celiac disease, multiple sclerosis, type 1 diabetes, and atherosclerosis [2,3,4,5,6,7]. Atherosclerosis is considered as a stress-related disease associated with cell activation and inflammation [8]. Activation and stress of multiple cell types within atherosclerotic plaque lesions are essential features of chronic inflammation, which may induce MICA/B expression on the surfaces of various cell types. Interestingly, MICA/B is upregulated on foam cells, one of the important cell types in the atherosclerosis process [5,9].

At present, the relationship between coronary artery disease (CAD) and various biological markers has been studied [10,11]. Extracellular vesicles (EVs) have been widely studied in many diseases. Physical characteristics and biochemical composition are applied to classify the EVs into two subtypes including exosomes and ectosomes. Accordingly, plasma membrane-derived EVs are categorized into ectosomes, which are also named as microparticles (MPs) or microvesicles [12]. Recently, circulating MPs from various cell origins have been extensively investigated in many diseases including CAD [13,14,15]. MPs are small vesicles released from various cells including platelets, red blood cells, leukocytes, granulocytes, monocytes, lymphocytes, and endothelial cells under conditions of cell activation, apoptosis, and stress. MPs are found in the plasma of healthy individuals and their amounts change in various clinical conditions. MPs can be recognized as both biological biomarkers and effectors of cell activation associated with pathological processes [16].

We, thus, hypothesized that activation and stress of various cell types during atherosclerosis played a crucial role in disease development. Under these conditions, MICA/B could appear on the cell surface and be released into blood circulation as MPs (MICA/B^+^ MPs). These parameters could be used as an indicator of vulnerable plaques leading to acute myocardial infarction (AMI). The present study aimed to investigate the expression of MICA/B on the surface of MPs from various cell origins in CAD patients presenting symptoms of AMI compared to age-matched disease control subjects. These results could provide novel findings revealing the association of MICA/B^+^ MPs and AMI. Thus, MICA/B^+^ MPs could be further applied as indicators of vulnerable plaques in patients with CAD, which subsequently leads to symptoms of AMI.

## 2. Materials and Methods

### 2.1. Study Population

Appropriate sample size was calculated to determine a difference between the means of two sample groups using the following formula (*n* = [2(Z_1−α/2_ + Z_1−β_)^2^ σ^2^] / δ^2^), where *n* = sample size, Z_1−α/2_ = 1.96 (alpha = 0.05), Z_1−β_ = 0.84 (power = 0.80), σ = variance of the observation in each group, and δ = meaningful difference in a population mean). Accordingly, total calculated study participants of AMI patients and controls were 27 for each group and subjects for ST-segment elevation myocardial infarction (STEMI) and non-STEMI (NSTEMI) were 18 for each group. A cross-sectional study was conducted among 51 randomly selected AMI subjects with ages greater than or equal to 30 years. Forty-six age-matched subjects without signs and symptoms of CAD, who had risk factors for CAD, were defined as the disease control group. In order to evaluate the association between MICA/B^+^ MPs and disease severity, AMI patients were divided into two groups including 26 STEMI and 25 NSTEMI admitted to Queen Sirikit Heart Center of the Northeast Hospital, Khon Kaen University, Thailand, during June 2015 to March 2018. The AMI included STEMI and NSTEMI following the criteria of the Third Universal Definition of Myocardial Infarction [17]. All medical records of AMI patients investigated by cardiologists were retrieved. This work was approved by the Khon Kaen University Ethics Committee for Human Research (HE571493/24-2-15) and consent forms were obtained from all participants. The study conformed to the principles outlined in the Declaration of Helsinki.

### 2.2. Blood Samples

Blood samples were obtained from patients within 1–2 h after the onset of AMI. Three milliliters of blood were collected in 3.2% trisodium citrate at a ratio of 9:1 (blood:anticoagulant) and placed at room temperature prior to plasma separation. Platelet-free plasma (PFP) was prepared within 1 h after blood collection by double centrifugation. Platelet-rich plasma (PRP) was first prepared by centrifugation at 2500× *g* for 15 min at room temperature. The second centrifugation of PRP at 2500× *g* for 15 min at room temperature was performed to obtain PFP. Subsequently, the plasma was immediately stored at −80 °C until analysis. Three milliliters of blood were collected to prepare serum for measuring blood chemistry parameters and high-sensitivity C reactive protein (hs-CRP). Complete blood count was performed using 3 mL of ethylenediaminetetraacetic acid (EDTA) blood.

### 2.3. MICA/B^+^ MPs’ Analysis

Expression of MICA/B on the surface of MPs that originated from endothelial cells (MICA/B^+^ EMPs), platelets (MICA/B^+^ PMPs), monocytes (MICA/B^+^ MMPs), neutrophils (MICA/B^+^ NMPs), and T lymphocytes (MICA/B^+^ TMPs) was investigated. Monoclonal antibodies including Annexin V-PE-Cy7 (Thermo Fisher Scientifics, Waltham, MA, USA), Alexa Fluor 488 mouse anti-human MICA/B (BioLegend, San Diego, CA, USA), PE mouse anti-human CD144 (Miltenyi Biotec, Bergisch Gladbach, Germany), APC mouse anti-human CD41a (BD Bioscience, San Jose, CA, USA), PE mouse anti-human CD14 (BD Bioscience, San Jose, CA, USA), APC mouse anti-human CD66b (Miltenyi Biotec, Bergisch Gladbach, Germany), and PerCP mouse anti-human CD3 (Miltenyi Biotec, Bergisch Gladbach, Germany) were used as specific markers for phosphatidylserine (PS), MICA/B, endothelial cells, platelets, monocytes, neutrophils, and T lymphocytes, respectively. Alexa Fluor 488 Mouse IgG2a (BioLegend, San Diego, CA, USA) were used as isotype control for anti-MICA/B.

**Figure 1:**
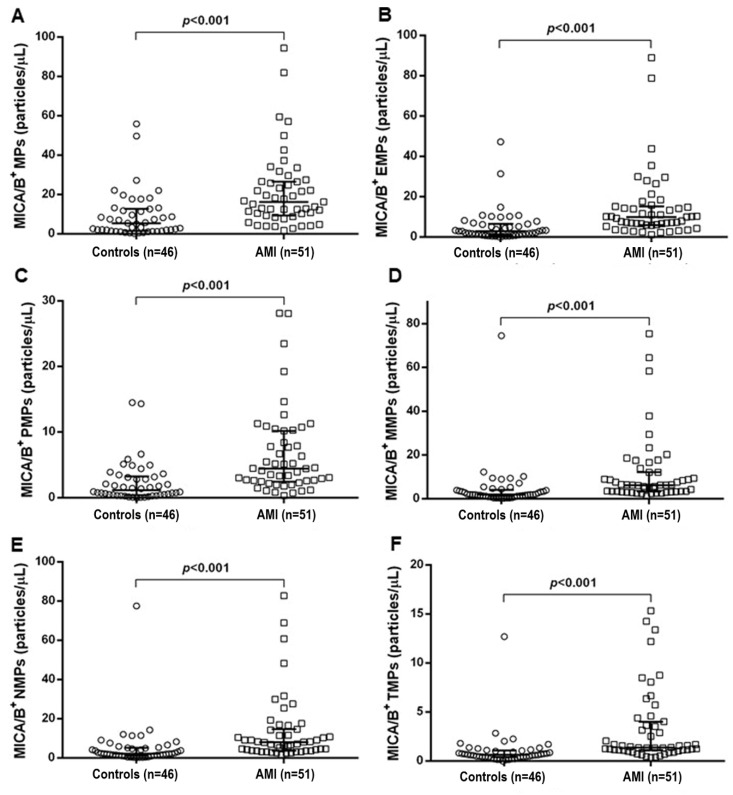
Scatter plots displaying the median with interquartile range absolute counts of MICA/B^+^ MPs from all cell types (**A**) and their distributions from various cell origins (**B**–**F**) in the controls and AMI patients. The numbers of MICA/B^+^ MPs from endothelial cells, platelets, monocytes, neutrophils, and T lymphocytes are shown in (**B**–**F**), respectively. MICA/B^+^ MPs from all types of cell origins were significantly increased in AMI compared to the controls. Please note that the scales are different among different sources of MPs. The major sources of MICA/B^+^ MPs were from endothelial cells, monocytes, and neutrophils. MICA/B: Major histocompatibility complex class I related chain A and B; MPs: Microparticles; EMPs: Endothelial-derived microparticles; PMPs: Platelet-derived microparticles; MMPs: Monocyte-derived microparticles; NMPs: Neutrophil-derived microparticles; TMPs: T lymphocyte-derived microparticles; AMI: acute myocardial infarction.

PFP samples were thawed in a water bath at 37 °C prior to analysis. The experiment was separated into two reactions. In the first reaction, 50 µL of PFP were mixed with 5 µL of Annexin V-PE-Cy7, Alexa Fluor 488 mouse anti-human MICA/B, PE mouse anti-human CD144, APC mouse anti-human CD41a, and 50 µL of diluted Annexin V-binding buffer solution containing calcium, which is essential for binding between Annexin V and PS on MPs. In the second reaction, 50 µL of PFP was mixed with 5 µL of Annexin V-PE-Cy7, Alexa Fluor 488 mouse anti-human MICA/B, PE mouse anti-human CD14, APC mouse anti-human CD66b, and PerCP mouse anti-human CD3, and 50 µL of diluted Annexin V-binding buffer solution. The mixtures were subsequently incubated for 15 min at room temperature in the dark. Subsequently, phosphate buffered saline, pH 7.4, was added to 300 µL of each reaction. The mixtures were performed in a 5-mL polystyrene round-bottom tube (BD Bioscience, San Jose, CA, USA). The 123count eBeads counting beads (Thermo Fisher Scientifics, Waltham, MA, USA) were used to calculate the absolute MP count. Then, the mixtures were immediately analyzed on a FACS Canto II flow cytometer (BD Bioscience, San Jose, CA, USA). The size of MPs was defined using 0.93-µM polystyrene beads (Spherotect, Lake Forest, IL, USA). The levels of MICA/B^+^ MPs were displayed as absolute numbers of MICA/B^+^ MPs from various cell origins (Figure 1) and were calculated using the following formula.

(1) $$\mathrm{Absolute}\;\mathrm{microparticle}\;\mathrm{count}\;\left(\mathrm{particles}/\left(µL\right)\right)=\frac{\mathrm{Number}\;\mathrm{of}\;\mathrm{microparticle}\;\mathrm{events}\;}{\mathrm{Number}\;\mathrm{of}\;\mathrm{counting}\;\mathrm{bead}\;\mathrm{events}}\times \frac{\mathrm{Number}\;\mathrm{of}\;\mathrm{known}\;\mathrm{counting}\;\mathrm{beads}/\mathrm{test}}{\mathrm{Test}\;\mathrm{volume}\;\left(µL\right)}$$

**Figure 2:**
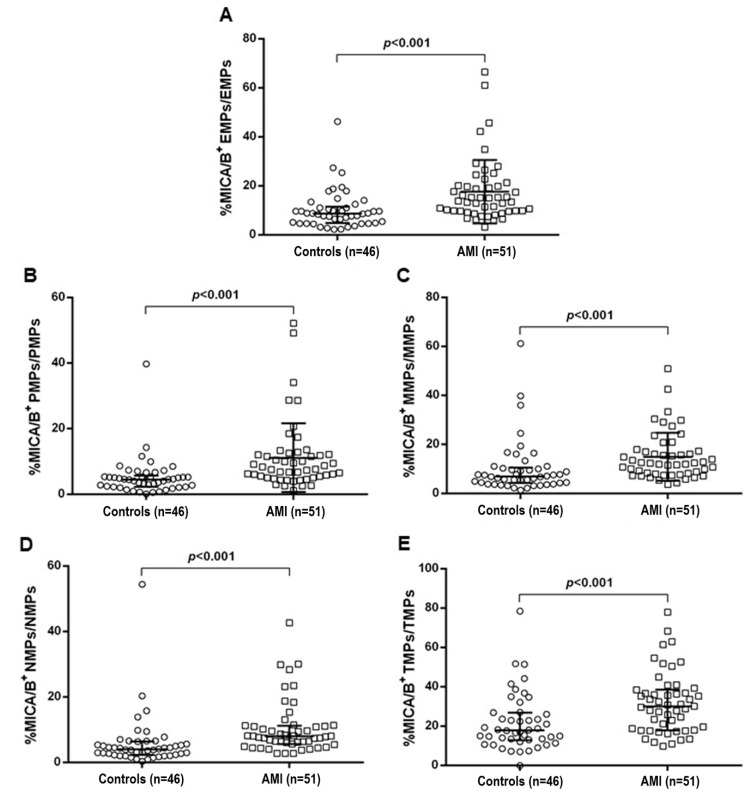
Scatter plots showing the median with interquartile range proportion of MICA/B^+^ MPs in each type of MPs in the control and AMI groups. The proportion of MICA/B^+^ MPs from endothelial cells, platelets, monocytes, neutrophils, and T lymphocytes are shown in (**A**–**E**), respectively. Proportions of MICA/B^+^ MPs of all cell types were elevated in AMI compared to the controls. MICA/B: Major histocompatibility complex class I related chain A and B; EMPs: Endothelial-derived microparticles; PMPs: Platelet-derived microparticles; MMPs: Monocyte-derived microparticles; NMPs: Neutrophil-derived microparticles; TMPs: T lymphocyte-derived microparticles; AMI: acute myocardial infarction.

The result was also shown as the proportion of MICA/B^+^ on each source of MPs, which was used to determine the intensity of cell stress and activation during the disease progression (Figure 2).

### 2.4. Statistical Analysis

Data were analyzed using SPSS version 17 (SPSS Inc., Chicago, IL, USA). Normal distribution of continuous variables was tested by the one-sample Kolmogorov–Smirnov test. Continuous values that were not in normal distribution were logarithm transformed before mean comparison. Independent-sample *t*-test was used to compare the means of each variable between two groups. Differences of proportions between two groups were tested using a Chi-square test. Binary logistic regression analysis including univariate and multivariate analysis adjusted for potential confounders was applied to determine the association between MICA/B^+^ MPs with AMI and disease severity. The relationship between continuous variables was determined by Spearman correlation. Statistical significance was defined as *p* < 0.05.

## 3. Results

### 3.1. Subject Characteristics

Demographic data of the study population are shown in Table 1. Ages of both groups were not significantly different whereas the proportions of male gender, diabetes mellitus, hypertension, dyslipidemia, smoking, and hs-CRP > 3 mg/L were significantly higher in patients than in the controls. Significant elevation of fasting blood sugar and hs-CRP was found in the AMI group whereas decreased levels of diastolic blood pressure, total cholesterol, and high-density lipoprotein cholesterol were observed. Demographic data of AMI patients are shown in Table 2. All parameters were not significantly different between NSTEMI and STEMI patients except the proportion of hypertension was higher in STEMI patients. The proportions of medications that can influence MP levels were also analyzed and revealed no significant differences between NSTEMI and STEMI patients (Table 3). In addition, the effect of statin and angiotensin converting enzyme inhibitor/angiotensin receptor blocker (ACEI/ARB) on MICA/B^+^ MP levels were evaluated. The results showed that MICA/B^+^ MP levels between the patients who had taken and not taken those drugs were not significantly different ((statin vs. without statin, 21.42 ± 15.43 vs. 21.60 ± 21.84, *p* = 0.536) and (ACEI/ARB vs. without ACEI/ARB, 22.47 ± 18.09 vs. 18.83 ± 22.58, *p* = 0.080)).

### 3.2. MICA/B^+^ MPs’ Analysis

**Figure 3:**
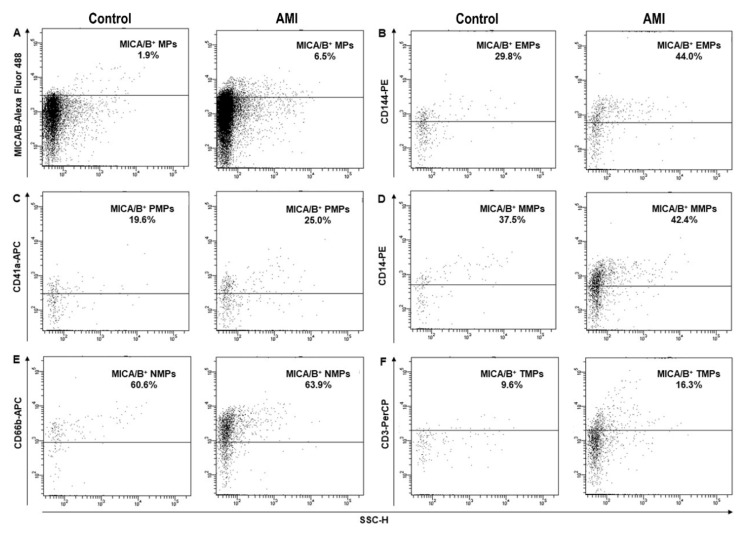
Flow cytometric analysis showing the levels of MICA/B^+^ MPs from all cell types in the disease control and AMI groups (**A**). MICA/B^+^ MPs are categorized according to their parent cells (**B**–**F**). The images of MICA/B^+^ MPs from endothelial cells, platelets, monocytes, neutrophils, and T lymphocytes are shown in (**B**–**F**), respectively. MICA/B: Major histocompatibility complex class I related chain A and B; MPs: Microparticles; EMPs: Endothelial-derived microparticles; PMPs: Platelet-derived microparticles; MMPs: Monocyte-derived microparticles; NMPs: Neutrophil-derived microparticles; TMPs: T lymphocyte-derived microparticles; AMI: acute myocardial infarction.

**Figure 4:**
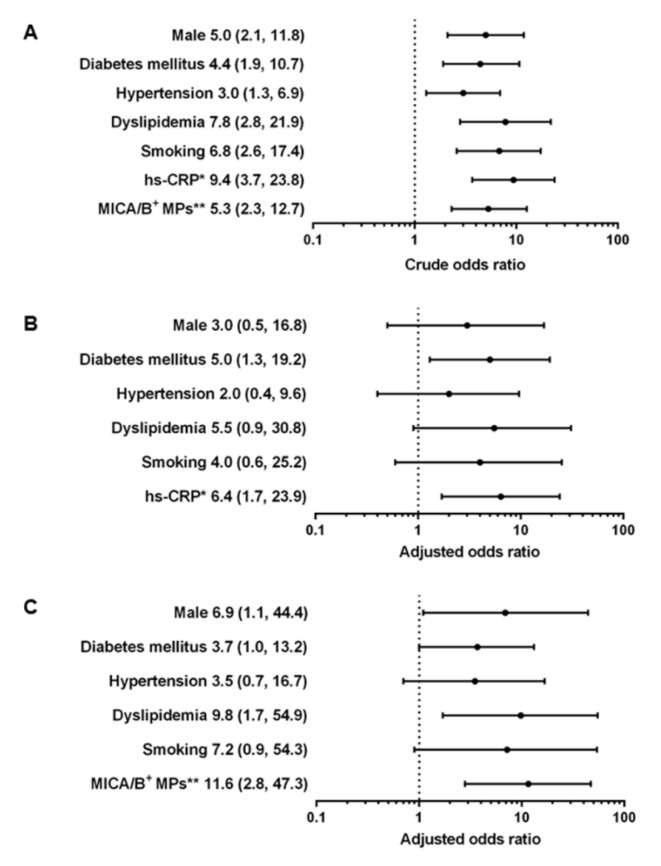
Crude odds ratios (**A**) and adjusted odds ratio with 95% confidence intervals demonstrating the association of hs-CRP (**B**) and MICA/B^+^ MPs (**C**) with the occurrence of AMI. Factors for adjusted odds ratios included male gender, diabetes mellitus, hypertension, dyslipidemia, and smoking. MICA/B^+^ MPs and hs-CRP were independently associated with AMI. * The level at 3 mg/L was used as the cutoff. ** The 50th percentile was used as the cutoff to distinguish high and low levels.

**Figure 5:**
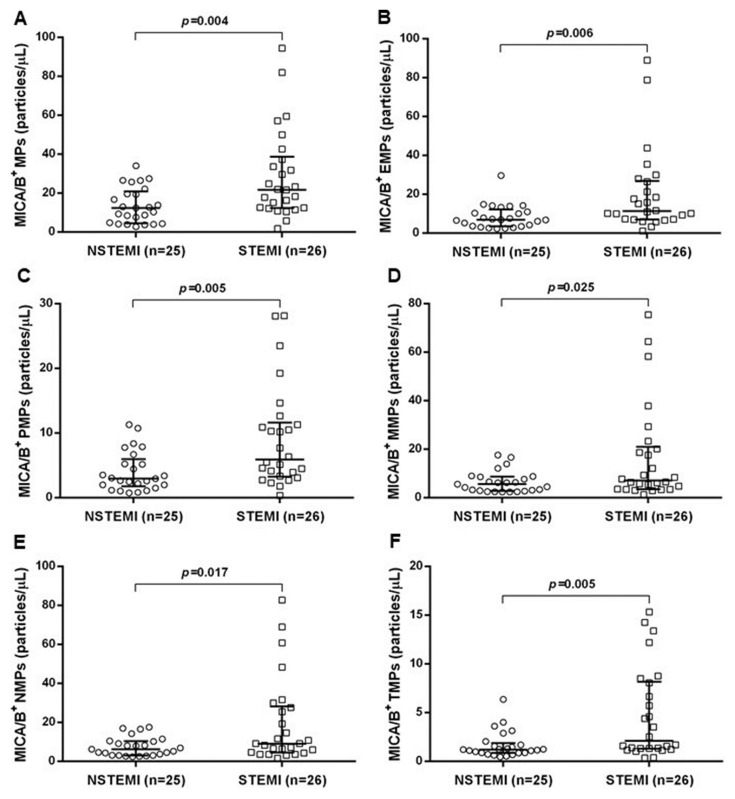
Scatter plots showing the median with interquartile range absolute counts of MICA/B^+^ MPs (**A**) and their distributions from various cell origins (**B**–**F**) in NSTEMI and STEMI patients. The numbers of MICA/B^+^ MPs from endothelial cells, platelets, monocytes, neutrophils, and T lymphocytes are shown in (**B**–**F**), respectively. MICA/B^+^ MPs from all types of cell origins were significantly increased in STEMI compared to NSTEMI. Please note that the scales are different among different sources of MPs. The major sources of MICA/B^+^ MPs were from endothelial cells, monocytes, and neutrophils. MICA/B: Major histocompatibility complex class I related chain A and B; MPs: Microparticles; EMPs: Endothelial-derived microparticles; PMPs: Platelet-derived microparticles; MMPs: Monocyte-derived microparticles; NMPs: Neutrophil-derived microparticles; TMPs: T lymphocyte-derived microparticles; STEMI: ST-segment elevation myocardial infarction; NSTEMI: non ST-segment elevation myocardial infarction.

**Figure 6:**
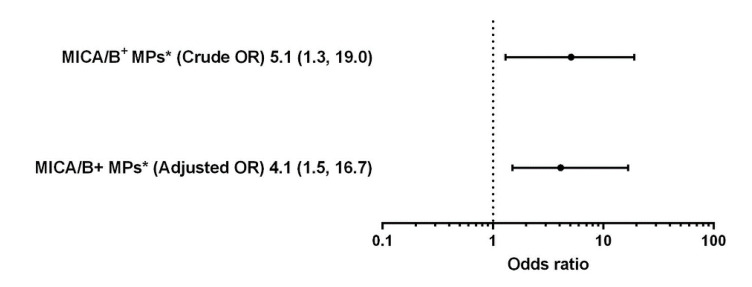
Crude odds ratio and adjusted odds ratio after adjustment for hypertension with 95% confidence intervals demonstrating the association of MICA/B^+^ MPs with the occurrence of STEMI. MICA/B^+^ MPs showed an independent association with STEMI. * The 50th percentile was used as the cutoff to distinguish high and low levels. MICA/B: Major histocompatibility complex class I related chain A and B; MPs: Microparticles.

**Figure 7:**
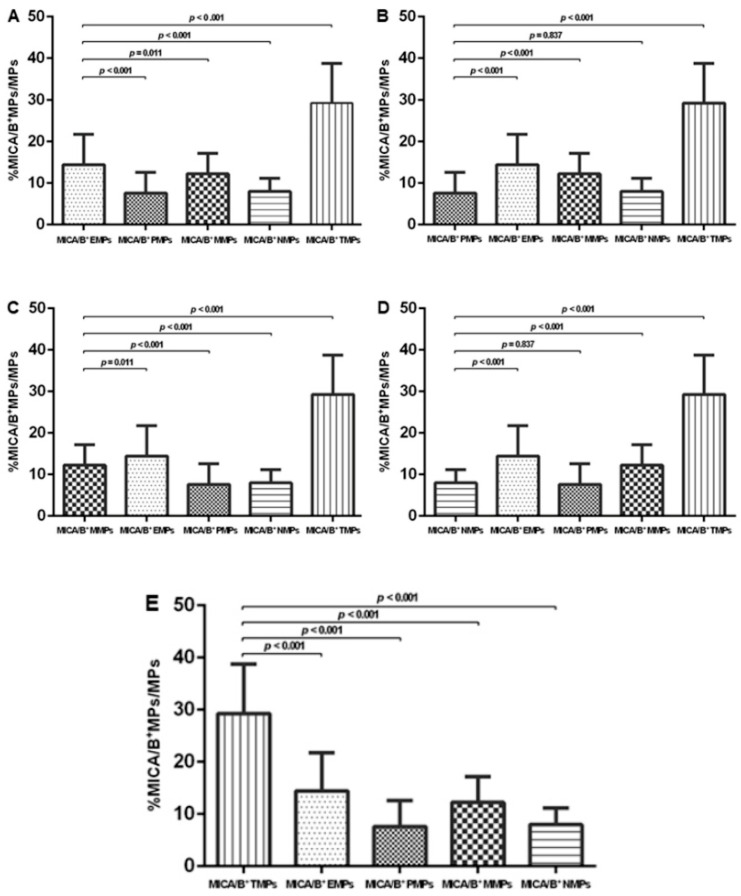
MICA/B expression on MPs from different cell origins. Comparisons among EMPs, PMPs, MMPs, NMPs, and TMPs were demonstrated in (**A**–**E**), respectively. MICA/B: Major histocompatibility complex class I related chain A and B; MPs: Microparticles; EMPs: Endothelial-derived microparticles; PMPs: Platelet-derived microparticles; MMPs: Monocyte-derived microparticles; NMPs: Neutrophil-derived microparticles; TMPs: T lymphocyte-derived microparticles.

**Figure 8:**
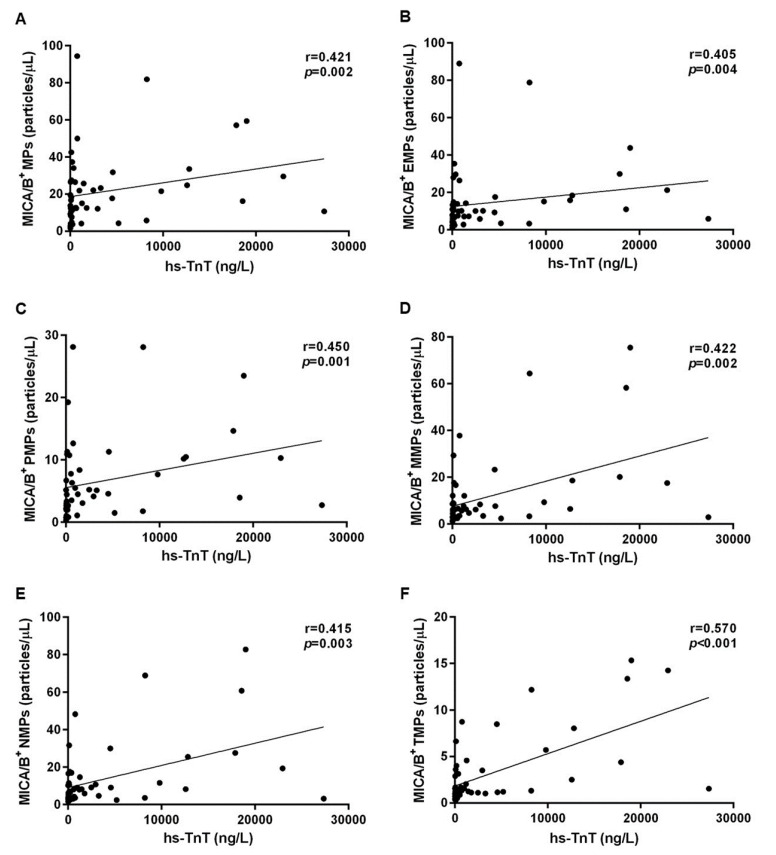
Correlations between MICA/B^+^ MPs from all cell types and hs-TnT in the study population (**A**). Correlations of MICA/B^+^ MPs from endothelial cells, platelets, monocytes, neutrophils, and T lymphocytes are shown in (**B**–**F**), respectively. MICA/B: Major histocompatibility complex class I related chain A and B; MPs: Microparticles; EMPs: Endothelial-derived microparticles; PMPs: Platelet-derived microparticles; MMPs: Monocyte-derived microparticles; NMPs: Neutrophil-derived microparticles; TMPs: T lymphocyte-derived microparticles; hs-TnT: high sensitivity-troponin T.

Levels of MICA/B^+^ MPs from all cell origins were significantly increased in the patients compared to those in the controls (Figure 1 and Figure 3). Similarly, the proportions of MICA/B^+^ MPs in each type of MPs were elevated in the patient group (Figure 2). A multivariate regression model adjusted for the traditional cardiovascular risk factors including male gender, diabetes mellitus, hypertension, dyslipidemia, and smoking [18] showed an independent association of hs-CRP and MICA/B^+^ MPs with AMI (Figure 4). Interestingly, this association was increased after combining with hs-CRP [odds ratio (OR) = 30.2; 95% confidence interval (CI) = 4.5, 202.0]. In addition, MICA/B^+^ MPs that originated from all parent cells were positively correlated with hs-CRP (Table 4). When the patients were divided into two subgroups according to the disease severity, the levels of MICA/B^+^ MPs that originated from all cell types were increased in patients with STEMI compared to the NSTEMI group (Figure 5). A multivariate regression model adjusted for hypertension, which was only a confounder associated with STEMI in this study, revealed the independent association of MICA/B^+^ MPs with the STEMI occurrence (Figure 6). The proportion of MICA/B expression on TMPs was significantly increased compared to other MPs (Figure 7). All types of MICA/B^+^ MPs were positively correlated with high sensitivity-troponin T (hs-TnT) levels, a necrotic marker for myocardial infarction (Figure 8).

## 4. Discussion

The present study is the first report of the association between MICA/B^+^ MPs considered as NKG2D ligands and AMI. MICA/B^+^ MPs from diverse cell origins in AMI patients were significantly elevated compared to the disease controls. Moreover, MICA/B^+^ MPs were an independent risk factor for AMI, and MICA/B^+^ MPs was more strongly associated with AMI than hs-CRP (Figure 4B,C). Synergic effect was demonstrated when MICA/B^+^ MPs was combined with hs-CRP. Accordingly, it was clearly established that MICA/B^+^ MPs were independently associated with the occurrence of AMI. In addition, the proportions of MICA/B^+^ MPs in all cell types of MPs were elevated in AMI. This suggested that the degree of cell activation leading to MICA/B expression in AMI was greater than that in the controls. In addition, all types of MICA/B^+^ MPs were positively correlated with hs-CRP, which is a biological marker of inflammatory diseases including atherosclerosis. It should be noted that the major MPs were from three sources, i.e., endothelial cells, monocytes, and neutrophils, suggesting the major contributions of activations of these cells in AMI.

Atherosclerosis is the underlying feature of CAD and AMI and has been speculated as the most common stress-related disease. Classically, inflammation plays a critical role in the initiation and progression of stress-related diseases. Proinflammatory substances induced by stress include CRP, interleukin (IL) -6, and tumor necrosis factor α [8]. Increases in inflammatory substances have been demonstrated in AMI patients [19]. Similarly, high levels of CRP were found among AMI patients in the present study. Atherosclerotic lesions from early disease stages to advanced lesions contain MPs that probably result from apoptosis, activation, and stress of cells in the plaque [20]. As a result, under conditions of chronic activation and stress in various cells, MICA/B can be presented on the cell surface because NKG2D ligands including MICA/B are induced in response to stress stimuli [21]. Consequently, MPs that express MICA/B are released from activated and stress cells during all stages of the disease. Therefore, MICA/B^+^ MPs may be markers of cell activation and stress. Moreover, they might be contributors to inflammation during atherosclerosis progression resulting from its role in stimulation of the NKG2D system.

Accordingly, the results of the present study revealed that MICA/B was present on all cell types and was subsequently released from cell surfaces as MICA/B^+^ MPs. Interestingly, the proportion of MICA/B expression on TMPs was significantly increased compared to other MPs (*p* < 0.001), albeit the amount of MICA/B^+^ TMPs was the smallest, suggesting the potency of T cells in the process. Alternatively, MICA/B^+^ TMPs may be the results of acquiring of MICA/B binding to NKG2D at the immune synapses in T cell activation, similar to NK cells [22]. At present, it is well known that T lymphocytes are the major players of adaptive immunity participating in atherosclerosis leading to AMI [23]. An increase in T cell activation has been shown in AMI. T helper 1 (Th1) cells are the major cell subset of T cells contributing to atherosclerosis [24]. Production of a large amount of interferon (IFN) γ is the crucial feature of Th1 activation found in AMI. Moreover, CD4 T cells from AMI patients display an increased response to T cell receptor stimulation [25]. Unfortunately, we did not classify the MP sources of the T cell subpopulation in this study. Thus, we do not know the contribution of T cell subpopulations producing MPs in AMI. Expectedly, experimental targets for T lymphocyte modulation are required to relieve the inflammatory response in atherosclerosis [26]. Hence, these evidences confirm that T cell activation in AMI encourages MICA/B expression in the cells.

Circulating MICA/B can be presented as membrane-bound and soluble forms. Therefore, MICA/B^+^ MPs may be included in the soluble MIC. Previous studies have shown that soluble MIC was associated with the occurrence and severity of diseases including cancer and inflammatory diseases [27,28,29]. Hence, MICA/B^+^ MPs, which are supposed to be included in the detection of soluble MIC in a serum sample, were found to be associated with AMI and the severity of disease in this study.

Previously, extensive studies on NKG2D ligands were performed in cancer along with a few in inflammatory diseases including Crohn′s disease and atherosclerosis [4,30]. Two reports confirming the association between MICA/B and atherosclerosis have been published. Firstly, Xia et al. [9] investigated the association between NKG2D ligands and diabetes mellitus and showed that type 2 diabetic patients had significantly higher levels of soluble MICA compared to healthy controls. Furthermore, they also revealed the presence of MICA/B in atherosclerotic aortic plaques of humans, where the ligands were expressed on macrophages and endothelial cells. Similarly, MICA/B^+^ MMPs and EMPs were abundant in this study. Interestingly, the NKG2D system was correlated with the development and progression of atherosclerotic plaques in mouse models. In addition, the study demonstrated that inhibition of NKG2D function could relieve inflammation by reducing the levels of many cytokines including IL-6, IFN-γ, and IL-1β. Secondly, Ikeshita et al. [5] demonstrated that MICA/B appeared in atherosclerotic plaque and was expressed on the surface of macrophages. Interestingly, the study showed that MICA/B could be specifically present in advanced atherosclerotic lesions including large necrotic cores, thin fibrous caps, or hemorrhagic lesions but not in stable plaques.

One of our major findings notes that MICA/B^+^ MPs from all cell origins were significantly increased in STEMI compared to NSTEMI patients, and that these parameters were independent risk factors for the occurrence of STEMI. Hypertension was the only risk factor used in the adjusted model because a significant difference in hypertension was observed between the NSTEMI and STEMI groups among the study subjects. It has been reported that levels of MPs were decreased by the effect of some vascular disease medications including statin, ACEI/ARB, aspirin, and clopidogrel [31]. Although the study subjects had taken these drugs, the proportion of NSTEMI and STEMI patients who were treated with these medications was not significantly different. Accordingly, the effect of medication on MP levels was not considered in this study.

In addition, MICA/B^+^ MPs were positively correlated with hs-TnT levels. It was thus confirmed that patients with STEMI had more inflammatory conditions than NSTEMI. Proinflammatory mediators including hs-CRP, IL-6, and granzyme B were significantly elevated in STEMI compared to NSTEMI [32,33]. Similarly, the present study demonstrated higher levels of hs-CRP in STEMI patients compared to NSTEMI patients but did not reach a significant level (*p* = 0.091). Interestingly, we found that MICA/B^+^ MPs from all cell types were positively correlated with the degree of AMI severity determined by the hs-TnT levels. The correlation between hs-TnT and inflammatory status in AMI examined by hs-CRP concentration has been investigated [34]. It is well known that TnT isoforms are extremely specific and sensitive to cardiac myocytes. Therefore, detection of hs-TnT in the blood stream is a highly specific marker for cardiac damage, resulting in different severities of myocardial injury in the AMI. Likewise, our results confirmed the correlation between hs-TnT and hs-CRP levels among AMI patients (r = 0.399, *p* = 0.005). Inflammatory status in AMI was found to be associated with MICA/B expression in various cells and correlated with the disease severity. Consequently, elevation of MICA/B^+^ MPs could reflect the intensity of inflammation, leading to more severe disease. Nevertheless, the main limitation of this study is a cross-sectional study design that could not demonstrate a utilization of MICA/B^+^ MPs as the early predictor of AMI symptom in CAD patients. A retrospective study in patients with stable CAD should be carried out.

## 5. Conclusions

To our knowledge, this is the first report investigating the association of MICA/B expressed on MPs from various cell origins in AMI. The results suggest that MICA/B^+^ MPs are associated with AMI and disease severity. Accordingly, MICA/B^+^ MPs could be mediators contributing to the pathological process of AMI. Alternatively, MICA/B^+^ MPs could be the results of cell activations and apoptosis in AMI. Nevertheless, they could represent inflammatory markers indicating the severity of AMI. As a result, MICA/B^+^ MPs could be further applied as an indicator of vulnerable plaques resulting in the symptoms of AMI and disease severity.

## Figures and Tables

**Table 1:** Demographic, clinical, and biochemical data of the study subjects.

Parameters	Control (*n* = 46)	AMI (*n* = 51)	*p*-Value
Age (years)	59.93 ± 7.50	61.86 ± 9.48	0.268
Gender (male: female)	14:32	35:16	<0.001
BMI (kg/m^2^)	25.41 ± 3.82	23.86 ± 3.83	0.051
SBP (mmHg)	134.41 ± 18.74	128.06 ± 23.57	0.148
DBP (mmHg)	80.71 ± 10.28	72.37 ± 13.84	0.001
Total cholesterol (mg/dL)	203.52 ± 44.47	167.28 ± 53.61	0.001
Triglyceride (mg/dL)	137.19 ± 90.49	142.81 ± 53.47	0.711
LDL-C (mg/dL)	128.28 ± 37.08	114.59 ± 48.47	0.127
HDL-C (mg/dL)	47.69 ± 15.57	40.14 ± 10.73	0.007
FBS (mg/dL)	95.80 ± 1.26	130.84 ± 1.39	<0.001
hs-CRP (mg/L)	1.51 ± 3.72	9.52 ± 4.77	<0.001
**Cardiovascular risk factors**			
Diabetes mellitus, *n* (%)	11 (23.91)	31 (60.78)	<0.001
Hypertension, *n* (%)	17 (36.95)	33 (64.71)	0.006
Dyslipidemia, *n* (%)	23 (50.00)	46 (90.20)	<0.001
Smoking, *n* (%)	8 (17.39)	29 (56.86)	<0.001
BMI ≥ 25 kg/m^2^, *n* (%)	25 (54.34)	21 (41.17)	0.195
hs-CRP > 3 mg/L, *n* (%)	13 (28.26)	41 (80.39)	<0.001

Values are mean ± SD or *n* (%), FBS and hs-CRP are expressed as geometric mean ± geometric standard deviation. BMI: Body mass index; SBP: Systolic blood pressure; DBP: Diastolic blood pressure; LDL-C: Low density lipoprotein-cholesterol; HDL-C: High density lipoprotein-cholesterol; FBS: Fasting blood sugar; hs-CRP: High sensitivity-C reactive protein.

**Table 2:** Demographic, clinical, and biochemical data of AMI patients.

Parameters	NSTEMI (*n* = 25)	STEMI (*n* = 26)	*p*-Value
Age (years)	62.80 ± 8.79	61.00 ± 10.21	0.495
Gender (male: female)	16:9	19:7	0.485
BMI (kg/m^2^)	23.98 ± 3.94	23.73 ± 3.78	0.818
SBP (mmHg)	134.16 ± 29.24	122.19 ± 14.71	0.075
DBP (mmHg)	73.40 ± 17.36	71.38 ± 9.57	0.613
Total cholesterol (mg/dL)	176.60 ± 47.35	159.03 ± 58.26	0.256
Triglyceride (mg/dL)	147.60 ± 57.04	138.57 ± 50.85	0.561
LDL-C (mg/dL)	116.60 ± 43.39	112.80 ± 53.35	0.785
HDL-C (mg/dL)	43.13 ± 12.94	37.50 ± 7.63	0.077
FBS (mg/dL)	124.09 ± 35.29	151.15 ± 58.92	0.061
hs-CRP (mg/L)	17.59 ± 26.74	29.05 ± 39.12	0.236
**Cardiovascular risk factors**			
Diabetes mellitus, *n* (%)	15 (60.00)	16 (61.53)	0.910
Hypertension, *n* (%)	12 (48.00)	21 (80.77)	0.005
Dyslipidemia, *n* (%)	23 (92.00)	23 (88.46)	0.671
Smoking, *n* (%)	12 (48.00)	17 (65.38)	0.348
BMI ≥ 25 kg/m^2^, *n* (%)	10 (40.00)	11 (42.20)	0.867
hs-CRP > 3 mg/L, *n* (%)	18 (72.00)	23 (88.46)	0.216

Values are mean ± SD. BMI: Body mass index; SBP: Systolic blood pressure; DBP: Diastolic blood pressure; LDL-C: Low density lipoprotein-cholesterol; HDL-C: High density lipoprotein-cholesterol; FBS: Fasting blood sugar; hs-CRP: High sensitivity-C reactive protein.

**Table 3:** Medications affecting MP levels that have been taken in AMI patients.

Medications	NSTEMI (*n* = 25)	STEMI (*n* = 26)	*p*-Value
Aspirin, *n* (%)	25 (100.00)	26 (100.00)	-
Clopidogrel, *n* (%)	25 (100.00)	26 (100.00)	-
Statin, *n* (%)	16 (64.00)	13 (50.00)	0.313
ACEI/ARB, *n* (%)	9 (36.00)	4 (15.38)	0.091

ACEI: Angiotensin converting enzyme inhibitor; ARB: Angiotensin receptor blocker.

**Table 4:** Correlations between MICA/B^+^ MPs and hs-CRP in the study population.

MPs	hs-CRP	
	r	*p*-Value
MICA/B^+^ MPs	0.336	0.001
MICA/B^+^ EMPs	0.362	<0.001
MICA/B^+^ PMPs	0.360	0.001
MICA/B^+^ MMPs	0.348	0.001
MICA/B^+^ NMPs	0.342	0.001
MICA/B^+^ TMPs	0.391	<0.001

hs-CRP: High sensitivity-C reactive protein; MICA/B: Major histocompatibility complex class I related chain A and B; MPs: Microparticles; EMPs: Endothelial-derived microparticles; PMPs: Platelet-derived microparticles; MMPs: Monocyte-derived microparticles; NMPs: Neutrophil-derived microparticles; TMPs: T lymphocyte-derived microparticles.
